# Diets high in selenium and isoflavones decrease androgen-regulated gene expression in healthy rat dorsolateral prostate

**DOI:** 10.1186/1477-7827-6-57

**Published:** 2008-11-24

**Authors:** Russell L Legg, Jessica R Tolman, Cameron T Lovinger, Edwin D Lephart, Kenneth DR Setchell, Merrill J Christensen

**Affiliations:** 1 Department of Nutrition, Dietetics and Food Science, Brigham Young University, Provo, UT 84602, USA; 2 Department of Physiology, Developmental Biology and Neuroscience, Brigham Young University, Provo, UT 84602, USA; 3 Department of Pediatrics, Children's Hospital Medical Center, Cincinnati, OH 45229, USA; 4 BYU Cancer Research Center, Brigham Young University, Provo, UT 84602, USA

## Abstract

**Background:**

High dietary intake of selenium or soybean isoflavones reduces prostate cancer risk. These components each affect androgen-regulated gene expression. The objective of this work was to determine the combined effects of selenium and isoflavones on androgen-regulated gene expression in rat prostate.

**Methods:**

Male Noble rats were exposed from conception until 200 days of age to diets containing an adequate (0.33-0.45 mg/kg diet) or high (3.33-3.45 mg/kg) concentration of selenium as Se-methylselenocysteine and a low (10 mg/kg) or high (600 mg/kg) level of isoflavones in a 2 × 2 factorial design. Gene expression in the dorsolateral prostate was determined for the androgen receptor, for androgen-regulated genes, and for Akr1c9, whose product catalyzes the reduction of dihydrotestosterone to 5alpha-androstane-3alpha, 17beta-diol. Activity of hepatic glutathione peroxidise 1 and of prostatic 5alpha reductase were also assayed.

**Results:**

There were no differences due to diet in activity of liver glutathione peroxidase activity. Total activity of 5alpha reductase in prostate was significantly lower (*p *= 0.007) in rats fed high selenium/high isoflavones than in rats consuming adequate selenium/low isoflavones. High selenium intake reduced expression of the androgen receptor, Dhcr24 (24-dehydrocholesterol reductase), and Abcc4 (ATP-binding cassette sub-family C member 4). High isoflavone intake decreased expression of Facl3 (fatty acid CoA ligase 3), Gucy1a3 (guanylate cyclase alpha 3), and Akr1c9. For Abcc4 the combination of high selenium/high isoflavones had a greater inhibitory effect than either treatment alone. The effects of selenium on gene expression were always in the direction of chemoprevention

**Conclusion:**

These results suggest that combined intake of high selenium and high isoflavones may achieve a greater chemopreventive effect than either compound supplemented individually.

## Background

Prostate cancer is the most frequently diagnosed cancer in American men and the second leading cause of all cancer deaths in men. The American Cancer Society estimates that there will be about 186,320 new cases of prostate cancer in the United States in 2008. About 28,660 men will die of this disease [1]. Because of its high occurrence and long latency period, prostate cancer is an ideal candidate for chemoprevention by dietary means. The Nutritional Prevention of Cancer Trial showed a significant reduction in prostate cancer incidence in men receiving a daily supplement of 200 μg selenium (Se) as selenized yeast [2,3]. Chan et al. [4], in their review of diet in the development and progression of prostate cancer, found the evidence more convincing for a protective effect of Se than for any other dietary component. The recently released report of the World Cancer Research Fund/American Institute for Cancer Research [5] summarized the available human data by stating "The evidence from cohort and case-control studies is consistent, with a dose-response relationship. There is evidence for plausible mechanisms. Foods containing selenium probably protect against prostate cancer."

Many possible molecular targets for Se have been identified [6-9] to account for its chemoprotective effects. Recent work has focused on the role in chemoprevention of selenoproteins [10], low molecular weight Se metabolites [11], and reactive oxygen species produced in Se metabolic pathways [12]. As a redox agent, Se can alter the conformation and activity of cellular proteins [13]. The redox regulation of transcription factors by Se has the potential to affect expression of multiple genes [14-17], and therefore the potential to affect risk for prostate cancer by multiple mechanisms simultaneously. These mechanisms include alteration of various aspects of steroid hormone metabolism [17,18].

Prostate cancer rates are low in Asian countries where the diet contains high levels of soy products [19] but high in countries characterized by consumption of a Western diet which is traditionally low in soy products [5,20-26]. Estrogenic isoflavones in soy are thought by many to account for this protective effect [27-31]. The protective effect of Se has been shown in populations consuming typical Western, low-soy diets. The purpose of this study was to determine whether Se and isoflavones may interact to reduce prostate cancer risk beyond that achieved by either dietary component alone.

Our working hypothesis is supported by the documented chemopreventive effects of Se that are due in part to its effects on androgen metabolism, androgen receptor (AR) expression and AR-regulated gene expression in the prostate. Androgens (e.g. testosterone) promote prostate cancer. Androgen ablation has traditionally been an important treatment strategy for prostate cancer. The anti-androgenic effects of Se are similar in many cases to those reported for isoflavones. The molecular mechanisms by which these dietary components exert their individual effects are the targets of current investigation, but little attention has focused on the potential chemopreventive efficacy of a supplemental combination of these two dietary components.

## Methods

### Animals and diets

All procedures related to animal care and use were approved by the Institutional Animal Care and Use Committee of Brigham Young University. Male and female Noble rats were purchased from Charles River Laboratories (Wilmington, MA) at thirty-five to forty-two days of age for use as breeders. This rodent strain has a low spontaneous incidence of grossly recognizable adenocarcinomas of the dorsal lobe of the prostate [32] but has been used in numerous studies of hormonally-induced prostate cancer [33] and has shown to be prone to steroid hormone reproductive tumors [34]. Four males and eight females were assigned to each dietary treatment. The two basal diets used were the Rodent Phytoestrogen Reduced I formulation of Zeigler Bros. (Zeigler Bros. Inc., Gardners, PA) which provides approximately 10 mg isoflavones/kg diet [35] and 0.45 mg Se/kg, and the Harlan-Teklad 8406 diet (Harlan-Teklad, Madison, WI) which provides roughly 600 mg isoflavones/kg and 0.33 mg Se/kg. The detailed composition of these two diets, as previously reported [36], is shown in Table 1. For 36 of the 43 nutrients listed, the difference in concentration is less than 50%, and for only one nutrient (Vitamin D) is the difference more than 2-fold. All nutrients were provided in both diets at levels that met or exceeded the minimum recommendations of the American Institute of Nutrition [37]. These two diets were fed with or without a supplement of 3 mg Se/kg diet as Se-methylselenocysteine, a naturally occurring food form of this mineral. The Se-methylselenocysteine used was a generous gift of Kelatron Corporation (Kelatron Corporation, Ogden, UT) and was incorporated into the diets by the manufacturers during formulation. For this chemical form of Se, the maximum tolerable dose, defined as "the dose that produced the first indication of a significant suppression in body weight", was previously reported to be 5.0 ppm [38]. The Se content of the high Se diets (3.33–3.45 ppm) used in this study was less than 70% of that value.

**Table 1:** Treatment diets

	**Unit**	**Harlan-Teklad 8604**	**Zeigler Bros. Phyto. Reduced I**
**Nutrient Composition**			
Protein	**%**	24.48	23.62
Fat	**%**	4.40	5.63
Fiber	**%**	3.69	2.38
Ash	**%**	7.84	6.40
Linoleic Acid	**%**	1.87	2.28

**Amino Acids**			
Arginine	**%**	1.53	1.11
Methionine	**%**	0.42	0.58
Cystine	**%**	0.37	0.27
Histidine	**%**	0.58	0.58
Isoleucine	**%**	1.24	1.17
Leucine	**%**	2.04	2.14
Lysine	**%**	1.46	1.41
Phenylalanine+tyrosine	**%**	1.84	2.05
Threonine	**%**	0.94	0.93
Tryptophan	**%**	0.29	0.25
Valine	**%**	1.26	1.35

**Minerals**			
Calcium	**%**	1.36	1.10
Phosphorus	**%**	1.01	0.92
Sodium	**%**	0.29	0.31
Chlorine	**%**	0.49	0.43
Potassium	**%**	1.04	0.55
Magnesium	**%**	0.28	0.17
Iron	mg/kg	352.14	354.26
Manganese	mg/kg	105.39	99.93
Zinc	mg/kg	82.87	61.69
Copper	mg/kg	24.42	12.69
Iodine	mg/kg	2.46	1.98
Cobalt	mg/kg	0.71	0.57
Selenium	mg/kg	0.33	0.45

**Vitamins**			
Vitamin A	IU/g	12.90	6.59
Vitamin D3	IU/g	2.40	7.13
Vitamin E	IU/g	90.18	51.25
Choline	mg/g	2.53	1.63
Niacin	mg/kg	63.42	69.47
Pantothenic acid	mg/kg	21.03	30.67
Pyridoxine (vitamin B6)	mg/kg	12.95	9.46
Riboflavin (vitamin B2)	mg/kg	7.85	6.66
Thiamine (vitamin B1)	mg/kg	27.95	16.35
Menadione (vitamin K3)	mg/kg	4.11	3.15
Folic acid	mg/kg	2.72	3.15
Biotin	mg/kg	0.39	0.29
Vitamin B12	mg/kg	51.20	47.92
Vitamin C	mg/kg	0.00	0.00

Ingredient list (first four) for the Harlan-Teklad 8604 diet: soybean meal, wheat middlings, flaked corn, ground corn Ingredient list (first four) for the Zeigler Bros. Phytoestrogen Reduced Diet I: corn, wheat, fish meal, wheat middlings

These diets provided Se and isoflavones in food forms and combinations naturally occurring in foodstuffs, rather than as semipurified diets with supplements of a single isoflavone and/or Se compound. The use of these diets, tested in pups whose exposure to the diets was begun at conception, was intended to imitate dietary conditions in Asian countries where prostate cancer rates are very low. While there were minor differences between basal diets in vitamin and mineral contents, the difference in isoflavone content was 60-fold. Likewise, the Se-supplemented diets provided a concentration of Se that was roughly 8–10 fold higher than the unsupplemented basal diets. Thus, it is highly likely that differences between diets in gene expression and other parameters were the result of differences in Se and isoflavone dietary concentrations, rather than minimal differences between diets in other nutrients.

Breeders consumed their respective diets for 30 days prior to mating. This ensured that pups used as subjects in this study would be exposed to their respective dietary treatments from conception. Eight offspring in each dietary group were weighed and sacrificed at 200 days of age. Blood was collected and dorsolateral prostate lobes and liver were dissected and weighed, then frozen in liquid nitrogen and stored at -80°C until analyzed. Many previous studies looking at androgen metabolism in the rodent have focused on the ventral prostate, or have not specified which lobes were examined. Much less attention has been given to the dorsolateral prostate lobes. However, the rodent ventral prostate has no clear homologous counterpart in the human prostate. The rodent dorsolateral prostate is the only prostate lobe comparable to the peripheral zone of the human prostate, from which most human tumors develop [39].

### Nutritional status assessment – serum analysis, enzyme activity

Analysis of serum isoflavone content was performed as described previously [40]. Briefly, phytoestrogen concentrations were analyzed for each dietary treatment group via gas chromatography/mass spectrometry (GC/MS). This was performed by liquid-solid extraction and liquid gel chromatographic techniques to isolate the phytoestrogen fractions using standards (internal controls) to validate the assay.

Due to limiting amounts of prostate tissue, activity of 5alpha-reductase in dorsal prostate lobes was assayed in just two dietary groups. We chose the two groups most likely to show a difference due to diet – rats fed the adequate Se/low isoflavone diet, and rats fed the high Se/high isoflavone diet. Analysis was performed as described in detail elsewhere [41]. Briefly, sections from dorsal prostate lobes were analyzed by TLC using [^3^H 1,2,6,7]testosterone as the substrate. Radioactivity was then measured in the reduced steroids [5alpha-androstane-3β, 17-dione, 5alpha-dihydrotestosterone and 5alpha-androstane-3alpha, 17 β-diol (3alpha-diol)] and activity rates were calculated. As a measure of Se status, activity of cellular Se-dependent glutathione peroxidase 1 (EC 1.11.1.9; GPX1) was assayed in cytosolic fractions from livers of all animals in each of the four diet treatments as previously described [42].

### Selection of genes for analysis

To select genes for examination in this study we reviewed the results of the data mining exercise of Zhang et al. [43]. By microarray analysis, they identified 422 AR-regulated genes in LNCaP human prostate cancer cells, and over 1000 Se-regulated genes in the same cell line. Comparison of the two lists revealed 92 genes regulated by both Se and androgen, of which 37 were reciprocally regulated. These authors also compared differences found in three independently published microarray analyses of gene expression in human prostate tumors compared to normal human prostate tissue. Over 1000 genes dysregulated in prostate cancer appeared in all three reports. Of the 37 genes reciprocally regulated by androgen and Se in LNCaP cells, 6 were among the genes shown by comparison of microarray analyses to be dysregulated in human prostate tumors. Thus, the genes selected for analysis in this study met three criteria: 1) they are dysregulated in human prostate cancer, 2) they are AR-regulated and 3) the androgen effect is opposed by Se. These genes included Abcc4 (ATP-binding cassette sub-family C member 4), Dhcr24 (24-dehydrocholesterol reductase), Facl3 (fatty acid CoA ligase 3), Gucy1a3 (guanylate cyclase alpha 3), and human kallikreins 2 and 3. Kallikreins 2 and 3 have no homologs in rats and were therefore not examined in this work. In addition to the four AR- and Se-regulated genes relevant in prostate cancer, for which homologs exist in rats, we examined expression of the AR itself and of Akr1c9 – the gene for the enzyme which catalyzes the reduction of dihydrotestosterone to its corresponding, less potent 5alpha-androstane-3alpha, 17beta-diol (commonly referred to as 3alpha-diol).

### Steady state mRNA analysis

Total RNA was isolated from rat dorsolateral prostates using TRIzol reagent (Invitrogen, Carlsbad, CA). Concentration and purity of RNA were determined spectrophotometrically, and RNA integrity was verified by gel electrophoresis. Equal quantities from each of three individual total RNA isolations within each dietary group were combined to form a total RNA pool for that group. Total RNA pools were reverse transcribed using random hexamers as primers. First strand cDNA was used as a template in quantitative PCR analysis (LightCycler, Roche, Mannheim, Germany) as we have previously described [44]. PCR Primers (Table 2) were designed using Accelrys DS gene 1.5 software (San Diego, CA). Optimum temperatures for primer annealing were determined experimentally for each primer pair using a range of annealing temperatures (RoboCycler, Stratagene, La Jolla, CA) followed by gel electrophoresis to confirm amplification of a single band of the expected size. For each gene, at least 3 LightCycler runs were performed. Each run included 3 replicates for each diet. Steady state mRNA levels for 18S rRNA were also quantified and used for normalization.

**Table 2:** PCR primer sequences

**Gene**	**Sequence (5' to 3')**
Abcc4	
- forward	TGCTTCCCAGACTCTGCACAAC
- reverse	AAGCAAGTCGTCCATGTGTCCG

Akr1c9	
- forward	CAAGTGCCTTTGAATGCTGAGCC
- reverse	ACACCCACTTCATGCCCAAGAC

AR	
- forward	AGAAAAAATCCCACATCCTGC
- reverse	CATCATTTCAGGAAAGTCCACG

Dhcr24	
- forward	TGCTGAACTCCATTGGCTGGAC
- reverse	TTGTGGGACGATGACTCGATGC

Facl3	
- forward	TCAGGCCAAGGCAAACTCCATTC
- reverse	TGCCAAAGCAAACTGGAGGAGG

Gucy1a3	
- forward	AAGCTGAAGGCAACCTTGGAGC
- reverse	TTTGTCCTTGCCAGAGCTGCTG

### Western blots

Proteins were isolated from dorsolateral prostate tissue from three rats in each dietary group. Equal quantities from each of three individual protein isolations within each dietary group were combined to form a protein pool for that group. Proteins (20 μg) were electrophoresed through denaturing SDS-PAGE gels (NuPAGE Novex 10% Bis-Tris gels, Invitrogen, Carlsbad, CA) and blotted to nitrocellulose membranes (Hybond ECL, GE Healthcare Life Sciences, Piscataway, NJ) as previously described [45]. Membranes were probed with antibodies to actin, AR, Facl3 (Acsl3), Abcc4 (Mrp4), (Novus Biologicals, Littleton, CO), Dhcr24 (Proteintech Group Inc., Chicago, IL), and Gucy1a3 (Santa Cruz Biotechnology, Inc., Santa Cruz, CA). A commercial source for Akr1c9 antibody was not found. Secondary antibodies were conjugated to infrared dyes (LI-COR Biosciences, Lincoln, NE) and detection was by direct infrared fluorescence (Odyssey Infrared Imaging System, LI-COR Biosciences, Lincoln, NE).

### Statistical analysis

Concentrations of amplified products for each replicate of each gene were calculated by the LightCycler software. To correct for possible minor differences in pipetting, initial cDNA concentrations, etc., the final concentration calculated by the LightCycler software for each replicate of a gene of interest in a dietary group was normalized by dividing by the calculated mean for all 18S rRNA replicates in that dietary group.

**Figure 1:**
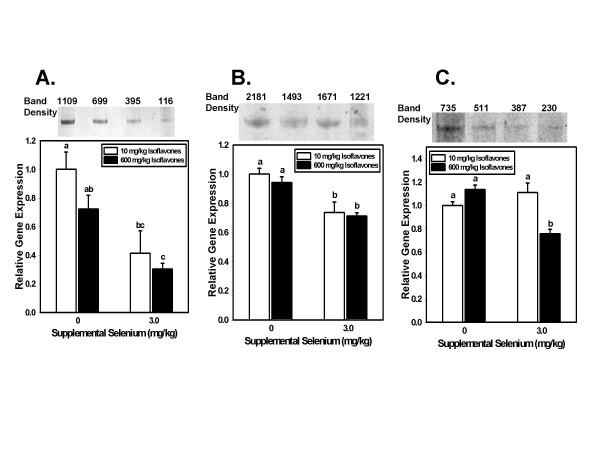
**Selenium-regulated gene expression**. Relative steady state levels (mean ± SEM) of mRNA in the dorsolateral prostates of rats fed diets providing 10 or 600 mg/kg isoflavones, and 0 or 3.0 mg/kg supplemental Se for 200 days, were determined by real time RT-PCR of total RNA. Analysis of variance was used to determine significance of the main effects of isoflavones and supplemental Se. Fisher's pairwise comparisons within the ANOVA were used to test differences between pairs of dietary groups. Bars not sharing a common superscript are significantly different (p < 0.05) by Fisher's pairwise comparisons. Values are expressed relative to the expression level of the 0 Se supplement-10 mg/kg isoflavone group. A. Androgen receptor gene expression. For the androgen receptor (AR) the main effect of supplemental Se was highly significant (p < 0.001) while the main effect of isoflavones was not (p = 0.253). B. Dhcr 24 gene expression. For Dhcr24 the reduction in gene expression due to high Se intake was statistically significant (p < 0.001) while the effect of isoflavones was not (p = 0.438). C. Abcc gene expression. For Abcc4 (Mrp4) the main effect of high supplemental Se intake in reducing gene expression was statistically significant (p = 0.025) while the reduction due to high isoflavone intake was not (p = 0.055).

**Figure 2:**
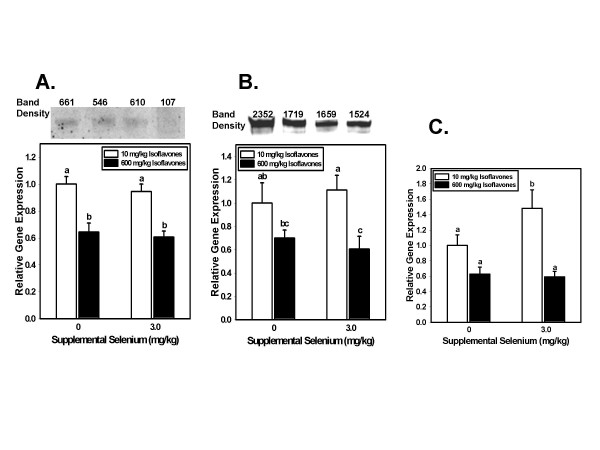
**Isoflavone-regulated gene expression**. Relative steady state levels (mean ± SEM) of mRNA in the dorsolateral prostates of rats fed diets providing 10 or 600 mg/kg isoflavones, and 0 or 3.0 mg/kg supplemental Se for 200 days, were determined by real time RT-PCR of total RNA. Analysis of variance was used to determine significance of the main effects of isoflavones and supplemental Se. Fisher's pairwise comparisons within the ANOVA were used to test differences between pairs of dietary groups. Bars not sharing a common superscript are significantly different (p < 0.05) by Fisher's pairwise comparisons. Values are expressed relative to the expression level of the 0 Se supplement-10 mg/kg isoflavone group. A. Gucy1a3 gene expression. For the Gucy1a3 gene the reduction due to high isoflavone intake was highly significant (p < 0.001) while the main effect of Se supplementation was not (p = 0.472). B. Facl3 gene expression. For Facl2 (Acsl3) isoflavones also significantly (p = .001) reduced gene expression while supplemental Se had no effect (p = 0.995). C. Akr1c9 gene expression. For rat aldo-keto reductase type 1 (Akr1c9) a significant (p < 0.001) reduction was seen in rats consuming high isoflavone diets, while the main effect of supplemental Se was not significant (p = 0.172).

For each gene, statistical analysis was performed on the 9–12 normalized replicates for each dietary treatment using analysis of variance (ANOVA), followed by Fisher's pairwise comparison to determine significance of differences between dietary groups (Minitab, State College, PA). In the process, a normalized mean value was calculated for each dietary group. Finally, to compare relative expression among the four dietary groups for each gene, the 18S-normalized mean for each of the other dietary groups was divided by the 18S-normalized mean of the adequate Se/low isoflavone group. These are the values shown in Figures 1 and 2.

Analysis of 5alpha-reductase data was done using a two-sample t-test to compare mean values for the adequate Se/low isoflavone diet to the high Se/high isoflavone diet (NCSS, Kaysville, UT). Data for GPX1 activity were also analyzed using ANOVA, followed by Fisher's pairwise comparison as described above.

## Results

### Body weight

There were no statistically significant effects of Se on body weight. This confirms the previous report [38] that the level of Se fed in the high Se diets in this study was not toxic to growing rats.

### Isoflavone analysis

As expected, serum isoflavone levels were significantly higher in rats fed the high isoflavone diets. Serum concentrations averaged 1254 ± 141 (mean ± SD) and 1659 ± 197 ng/mL in rats consuming high isoflavone diets, with and without a 3.0 mg/kg Se supplement, respectively (p < 0.05). In contrast, serum concentrations in rats fed the low isoflavone diets averaged 22.0 ± 2.3 and 24.6 ± 0.1 ng/mL in animals with and without the Se supplement, respectively. The total serum isoflavone concentrations in rats fed 600 mg/kg were similar to those of adults living in Asia while the levels in rats fed only 10 mg/kg were comparable to those in persons consuming typical Western diets [24,46].

### Enzyme activity

**Figure 3:**
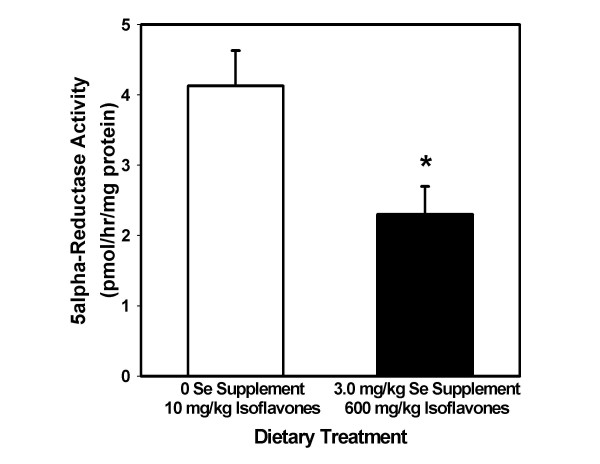
**Rat dorsolateral prostate 5alpha-reductase activity**. Activity (mean ± SEM) of 5alpha-reductase was determined in dorsolateral prostates of rats fed the 0 Se supplement-10 mg/kg phytoestrogen diet, and compared with activity in rats fed the 3.0 mg/kg Se supplement-600 mg/kg phytoestrogen diet. *The combination of high levels of both dietary components significantly reduced enzyme activity (p < .001).

There were no significant differences due to diet in the activity of hepatic GPX1. This was expected since all diets provided a concentration of Se higher than needed to maximize activity of this enzyme in rat liver. Mean activities in the four dietary groups ranged from 453–567 mU/mg protein, which are comparable to previously published values for GPX1 activity in Se-adequate rat liver [42,47]. Figure 3 shows that rats fed the high Se-high isoflavone diet had a lower total 5alpha-reductase activity (*p *= 0.001) when compared to rats fed diets low in Se and isoflavones.

### Gene expression

Figures 1 and 2 show genes for whose expression the main effect of Se (Figure 1) or of isoflavones (Figure 2) was statistically significant. Above each bar graph depicting steady state mRNA levels is shown an inset of a Western blot probed with an antibody to that protein. Results of densitometric scanning of band intensities are shown above each inset. As noted above, we were unable to find a commercial source for an antibody to rat Akr1c9. Stripping each blot and probing with an antibody to actin showed no significant differences in gel loading.

In agreement with findings in cultured cells, high Se intake by rats in this study dramatically reduced expression of the AR in the dorsolateral prostate (Figure 1A). Somewhat surprisingly, the main effect of isoflavones was not statistically significant.

The Dhcr24 gene codes for an antiapoptotic protein which inhibits caspase 3. Rats consuming high Se diets had significantly lower expression of this gene (p < 0.001) than animals fed diets without the Se supplement (Figure 1B). There was no significant effect of isoflavones on Dhcr24 gene expression (p = 0.438).

Abcc4 (also called Mrp4) is a member of the multidrug resistance protein family. Its down-regulation may enhance the efficacy of anticancer drugs. The main effect of high supplemental Se intake in reducing Abcc4 gene expression was statistically significant (p = 0.025) (Figure 1C). High isoflavone intake also modestly decreased gene expression, but that main effect was not statistically significant (p = 0.055). This was the only gene for which the combination of high Se and high isoflavones had a more significant effect than either compound alone, or any other combination.

High isoflavone intake significantly decreased the expression of the Gucy1a3 gene (p < 0.001) (Figure 2A). The product of this gene catalyzes conversion of GTP to the second messenger cyclic GMP, which regulates the activity of a variety of protein kinases, phosphodiesterases, and ion channels. The main effect of Se on Gucy1a3 gene expression was not significant (p = 0.472).

Facl3 (fatty acid CoA ligase 3, also called acyl CoA synthetase 3, Ascl3) is associated with fatty acid-induced apoptosis. Ironically, in the cell culture work of Zhang et al., androgen increased expression of this potentially protective gene while Se decreased its expression. In this work, in which rats were rats fed different levels of Se and isoflavones, high isoflavones significantly down-regulated Facl3 (p = 0.001), but Se had no significant effect (p = 0.995) (Figure 2B).

Expression of the Akr1c9 gene – the rat homolog of the human gene whose product reduces dihydrotestosterone to its 3alpha-diol – is decreased in prostate cancer. Somewhat surprisingly, expression of this gene was also decreased by high isoflavone intake (Figure 2C). The main effect of treatment with 3.0 mg/kg Se on expression of Akr1c9 was not statistically significant, although expression in the low isoflavone/high Se group was significantly greater than all other dietary groups.

## Discussion

The AR plays a role in the growth of both healthy prostate and prostate tumors. Studies of cultured prostate cancer cells show an inhibitory effect of Se on AR gene expression [48-51] and various mechanisms have been proposed [52]. Recently Lee et al. reported that Se injections decreased AR gene expression in LNCaP xenographs in nude mice [53]. In this work, we report for the first time a significant decrease in AR expression in the prostates of healthy animals *fed *a nutritionally relevant form of Se. This confirms findings in the xenograft and in vitro models and suggests a possible mechanism for the protective effects of dietary Se against prostate cancer.

Previous studies in cultured cells and animal models have also shown that high isoflavone treatments reduce AR gene expression [54-57]. That effect was not seen in this work. However, most previous studies have employed isolated, individual soy isoflavones (most often genistein) at widely varying (and sometimes unphysiologic/pharmacologic) levels (50). In some cases, effects of isolated genistein on cultured cancer cells have been shown to be at variance with the observed chemoprotective effects of soy product consumption by humans [58-60]. In this work isoflavones were provided in stock diets formulated from foodstuffs naturally high or low in phytoestrogens, which provide isoflavones in their naturally occurring dietary blend, as is the case for diets consumed by human populations. It should also be noted that previous animal experiments have either not specified which lobe of the prostate was studied or have focused on the ventral lobes, which have a markedly different gene expression pattern for AR than the dorsolateral prostate lobes [61]. This study focused on the dorsolateral lobes of the prostate, which are the most similar to the human prostate peripheral zone, in which the majority of prostate tumors develop.

The enzyme 5alpha-reductase uses NADPH to reduce testosterone to the more potent hormone dihydrotestosterone (DHT). El-Bayoumy et al. found no effect on activity of 5alpha-reductase in healthy men given Se-enriched yeast [62]. Likewise, Weber et al. found the activity of the enzyme to be unaffected in rats fed high amounts of isoflavones [36]. The observed decrease in 5alpha-reductase activity in this work suggests that combining Se and isoflavones in the diet may produce an effect greater than previously seen with either treatment alone.

The Dhcr24 gene codes for the enzyme 3beta-hydroxysterol-delta24 reductase (alias seladin-1) which catalyzes the conversion of desmosterol to cholesterol and regulates responses to oncogenic and oxidative stimuli [63]. Through its role in cholesterol biosynthesis, Dhcr24 has been reported to act as an antiapoptotic factor [64] by functioning in the activation and maintenance of the Akt-Bad cell survival cascade [65] and the Ras signaling pathway [66]. Associations between dietary Se, isoflavones, and Dhcr24 metabolism have not been previously explored. In this work high dietary Se, but not isoflavones, decreased the expression of the Dhcr24 gene. This suggests another mechanism by which Se, through the decreased expression of this antiapoptotic protein, may be chemopreventive.

ATP-binding cassette, sub-family C, member 4 (Abcc4/Mrp4) is a multiple drug resistance protein that mediates the efflux out of cells of nucleotide and nucleoside analogues which are an important class of therapeutic agents [67]. In prostate cancer, Abcc4 expression is up-regulated. In our study, rats fed high dietary Se experienced a decrease in Abcc4 gene expression. In addition, the combination of high Se/high isoflavone intake achieved an effect greater than the use of either compound individually. This suggests that dietary intervention may enhance the therapeutic effect of various anti-cancer drugs [50].

Guanylate cyclase 1 alpha 3 (Gucy1a3) catalyzes the conversion of GTP to the second messenger cyclic guanosine 3',5'-monophosphate (cGMP), which regulates the activity of protein kinases, phosphodiesterases, and ion channels [43]. It is consistently overexpressed in prostate cancer. In LNCaP cells, cGMP activates various forms of mitogen-activated protein kinases (MAPK) [68], which leads to phosphorylation of the AR [69] and subsequent DNA binding. Previous work in LNCaP cells showed that isoflavones down-regulate MAPK [70]. A decrease in Gucy1a3 leading to decreases in cGMP and MAPK activity may correspond to a decrease in AR phosphorylation. Our finding of a decreased Gucy1a3 expression in rats fed high isoflavones suggests a possible mechanism for the previously reported results of isoflavones' effect on MAPK, and is consistent with a chemopreventive effect due to decreased AR activation.

Acyl-CoA synthetase long-chain family member 3 (Facl3/Acsl3) catalyzes the initial reaction of fatty acid metabolism to produce Acyl-CoA and is associated with fatty acid-induced apoptosis. Interestingly, Facl3 is upregulated by androgen [51] and vitamin D_3 _[71] acting through an AR-mediated pathway in LNCaP cells. In the same cell line Se was shown to decrease Facl3 expression [43]. This is consistent with Se's established effects in cell culture on AR expression. In contrast, in rats fed varying combinations of Se and isoflavones, only isoflavone consumption was associated with a significant decrease in the expression of this gene. This is the first report of a relationship between isoflavone consumption and Facl3 gene expression. Waku et al. showed that Facl3 inhibits the thyroid receptor [72], which is a member of the nuclear hormone receptor family. The effects of Facl3 on the AR and other members of the nuclear receptor family remain to be explored.

The rodent enzyme Akr1c9 is a member of the aldo-keto reductase superfamily. The physiologic function of Akr1c9 is to catalyze the reversible reduction of dihydrotestosterone to androstanediol [alpha-diol] [73]. In this way, Akr1c9 regulates intracellular hormone concentration and ligand availability for the AR. A key step in AR activation is ligand binding of DHT. An increase in Akr1c9 mRNA expression would be consistent with chemoprevention by decreasing DHT availability for the AR. However, in rats fed diets containing high levels of isoflavones, there was an unanticipated decrease in Akr1c9 mRNA levels. One possible explanation is that the Akr1c9 gene is positively regulated by steroid hormone receptor activation [74]. There may be a correlation between the observed decrease in AR mRNA levels in this work and the decreased expression of Akr1c9, an AR-regulated gene.

**Figure 4:**
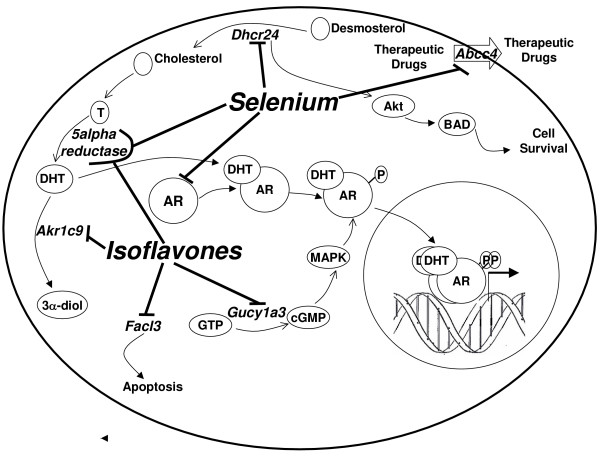
**Proposed model and summary**. The statistically significant main effects of Se and isoflavones on gene expression are shown, as well as the significant combined effect of high Se-high isoflavone intake on 5alpha reductase activity. The model shows the role of each gene product in metabolic pathways and the relationship of those pathways to one another. Elucidation of effects on gene expression in this study suggests molecular mechanisms for the individual effects of chemopreventive Se and isoflavones individually, and the potential for an enhanced protective effect by combining the two dietary treatments.

Figure 4 summarizes our results and is a proposed model for the molecular mechanisms by which Se and isoflavones may exert their previously demonstrated inhibitory effects on prostate cancer development and progression. For those genes whose expression was significantly altered by high Se intake, the effects of diet were consistent with cancer chemoprevention. In contrast, in two of the three cases where high isoflavone intake had a statistically significant effect on gene expression, the effect was in the opposite direction of that associated with protection. It should be noted that the four genes from the data mining experiment selected for study in this work were chosen based on their relevance in prostate cancer and on their responsiveness to Se and androgen, not isoflavones. The effects of isoflavones on expression of these genes strongly suggest that foods containing isoflavones may be chemopreventive even though not every effect on expression of individual genes is in the direction of protection.

## Conclusion

In summary, this is the first report of AR down-regulation by dietary Se in healthy animals. The dietary effects observed suggest mechanisms for Se's previously demonstrated chemopreventive properties. In addition, the observations that a) high Se and high isoflavones each had significant effects on expression of different genes; and b) the combination of high Se and high isoflavone intake significantly reduced Abcc4 gene expression, compared to the other three dietary groups, suggest that a combination of these two dietary components consumed at high levels may achieve an even greater chemopreventive effect than high consumption of either compound alone.

## Competing interests

One of the co-authors (EDL) serves as an adviser to the editorial board of Reproductive Biology and Endocrinology.

## Authors' contributions

RLL participated in study design, quantitated gene expression, and assisted in manuscript preparation. JRR and CTL were responsible for animal husbandry (breeding, feeding, weighing, sacrifice, dissection, etc.), assayed glutathione peroxidase activity, and assisted in quantitating gene expression. EDL assisted in study design, conducted 5alpha-reductase assays, and contributed to manuscript preparation. KDRS was responsible for assay of blood levels of isoflavones. MJC conceived the study, participated in study design, performed statistical analysis, and helped draft the manuscript. All authors read and approved the final manuscript.

## References

[B1] Jemal A, Siegel R, Ward E, Hao Y, Xu J, Murray T, Thun MJ (2008). Cancer statistics, 2008. CA Cancer J Clin.

[B2] Clark LC, Combs GF, Turnbull BW, Slate EH, Chalker DK, Chow J, Davis LS, Glover RA, Graham GF, Gross EG, Krongrad A, Lesher JL, Park HK, Sanders BB, Smith CL, Taylor JR (1996). Effects of selenium supplementation for cancer prevention in patients with carcinoma of the skin. A randomized controlled trial. Nutritional Prevention of Cancer Study Group. JAMA.

[B3] Duffield-Lillico AJ, Dalkin BL, Reid ME, Turnbull BW, Slate EH, Jacobs ET, Marshall JR, Clark LC (2003). Selenium supplementation, baseline plasma selenium status and incidence of prostate cancer: an analysis of the complete treatment period of the Nutritional Prevention of Cancer Trial. BJU Int.

[B4] Chan JM, Gann PH, Giovannucci EL (2005). Role of diet in prostate cancer development and progression. J Clin Oncol.

[B5] World Cancer Research Fund/American Institute for Cancer Research (2007). Food, Nutrition, Physical Activity, and the Prevention of Cancer: a Global Perspective.

[B6] Combs GF (2004). Status of selenium in prostate cancer prevention. Br J Cancer.

[B7] Klein EA (2005). Chemoprevention of prostate cancer. Crit Rev Oncol Hematol.

[B8] Klein EA, Thompson IM (2004). Update on chemoprevention of prostate cancer. Curr Opin Urol.

[B9] McCarty MF (2004). Targeting multiple signaling pathways as a strategy for managing prostate cancer: multifocal signal modulation therapy. Integr Cancer Ther.

[B10] Hu Y, Benya RV, Carroll RE, Diamond AM (2005). Allelic loss of the gene for the GPX1 selenium-containing protein is a common event in cancer. J Nutr.

[B11] Lu J, Jiang C (2005). Selenium and cancer chemoprevention: hypotheses integrating the actions of selenoproteins and selenium metabolites in epithelial and non-epithelial target cells. Antioxid Redox Signal.

[B12] Spallholz JE, Palace VP, Reid TW (2004). Methioninase and selenomethionine but not Se-methylselenocysteine generate methylselenol and superoxide in an in vitro chemiluminescent assay: implications for the nutritional carcinostatic activity of selenoamino acids. Biochem Pharmacol.

[B13] Ganther HE (1999). Selenium metabolism, selenoproteins and mechanisms of cancer prevention: complexities with thioredoxin reductase. Carcinogenesis.

[B14] Christensen MJ, Olsen CA, Hansen DV, Ballif BC (2000). Selenium regulates expression in rat liver of genes for proteins involved in iron metabolism. Biol Trace Elem Res.

[B15] Kendall SD, Christensen MJ (1997). Selenium deficiency decreases expression of the genes for transthyretin and citrate transport protein in rat liver. Nutr Res.

[B16] Nelson KK, Bacon B, Christensen MJ (1996). Selenite supplementation decreases expression of MAZ in HT29 human colon adenocarcinoma cells. Nutr Cancer.

[B17] Yang Q, Christensen MJ (1998). Selenium regulates gene expression for estrogen sulfotransferase and alpha 2U-globulin in rat liver. J Steroid Biochem Mol Biol.

[B18] Hansen DV, Nielsen DR, Felin EM, Johnson JI, Christensen MJ (2000). Regulation of gene expression in rat prostate by selenium. FASEB J.

[B19] Sarkar FH, Li Y (2003). Soy isoflavones and cancer prevention. Cancer Invest.

[B20] Adlercreutz H (1997). Evolution, nutrition, intestinal microflora, and prevention of cancer: a hypothesis. Proc Soc Exp Biol Med.

[B21] Adlercreutz H (1998). Epidemiology of phytoestrogens. Baillieres Clin Endocrinol Metab.

[B22] Adlercreutz H, Mazur W, Bartels P, Elomaa V, Watanabe S, Wahala K, Landstrom M, Lundin E, Bergh A, Damber JE, Aman P, Widmark A, Johansson A, Zhang JX, Hallmans G (2000). Phytoestrogens and prostate disease. J Nutr.

[B23] Barnes S (1998). Evolution of the health benefits of soy isoflavones. Proc Soc Exp Bio Med.

[B24] Griffiths K, Denis L, Turkes A, Morton PS (1998). Phytoestrogens and diseases of the prostate gland. Baillieres Clin Endocrinol Metab.

[B25] Mazur W, Adlercreutz H (2000). Overview of naturally occurring endocrine-active substances in the human diet in relation to human health. Nutrition.

[B26] Setchell KDR (1998). Phytoestrogens: biochemistry, physiology and implications for human health of soy isoflavones. Am J Clin Nutr.

[B27] Adlercreutz H, Mazur W (1997). Phyto-oestrogens and Western diseases. Ann Med.

[B28] Knight DC, Eden JA (1996). A review of the clinical effects of phytoestrogens. Obstet Gynecol.

[B29] Kurzer MS, Xu X (1997). Dietary phytoestrogens. Annu Rev Nutr.

[B30] Murkies AL, Wilcox G, Davis SR (1998). Phytoestrogens-review. J Clin Endocrinol Metab.

[B31] Setchell KDR, Cassidy A (1999). Dietary isoflavones-biological effects and relevance to human health. J Nutr.

[B32] Noble RL (1977). The development of prostatic adenocarcinoma in Nb rats following prolonged sex hormone administration. Cancer Res.

[B33] Ouyang XS, Wang X, Lee DT, Tsao SW, Wong YC (2001). Up-regulation of TRPM-2, MMP-7 and ID-1 during sex hormone-induced prostate carcinogenesis in the Noble rat. Carcinogenesis.

[B34] Yuen M-T, Leung L-K, Wang J, Wong Y-C, Chan FL (2005). Enhanced induction of prostatic dysplasia and carcinoma in Noble rat model by combination of neonatal estrogen exposure and hormonal treatments at adulthood. Int J Oncol.

[B35] Lephart ED, Rhees RW, Setchell KDR, Bu LH, Lund TD (2003). Estrogens and phytoestrogens: brain plasticity of sexually dimorphic brain volumes. J Steroid Biochem Mol Biol.

[B36] Weber KS, Setchell KDR, Stocco DM, Lephart ED (2001). Dietary soy-phytoestrogens decrease testosterone levels and prostate weight, without altering LH, prostate 5alpha-reductase or testicular StAR levels in adult male Sprague-Dawley rats. J Endocrinol.

[B37] Reeves PG, Nielsen FH, Fahey GC (1993). AIN-93 purified diets for laboratory rodents: final report of the American Institute of Nutrition ad hoc writing committee on the reformulation of the AIN-76A rodent diet. J Nutr.

[B38] Medina D, Thompson H, Ganther H, Ip C (2001). Se-methylselenocysteine: a new compound for chemoprevention of breast cancer. Nutr Cancer.

[B39] Imasato Y, Onita T, Moussa M, Sakai H, Chan FL, Koropatnick J, Chin JL, Xuan JW (2001). Rodent PSP94 gene expression is more specific to the dorsolateral prostate and less sensitive to androgen ablation than probasin. Endocrinology.

[B40] Setchell KD, Brown NM, Desai P, Zimmer-Nechemias L, Wolfe BE, Brashear WT, Kirschner AS, Cassidy A, Heubi JE (2001). Bioavailability of pure isoflavones in healthy humans and analysis of commercial soy isoflavone supplements. J Nutr.

[B41] Lephart ED, Andersson S, Simpson ER (1990). Expression of neural 5 alpha-reductase messenger ribonucleic acid: comparison to 5 alpha-reductase activity during prenatal development in the rat. Endocrinology.

[B42] Christensen MJ, Cammack PM, Wray CD (1995). Tissue specificity of selenoprotein gene expression in rats. J Nutr Biochem.

[B43] Zhang H, Dong Y, Zhao H, Brooks JD, Hawthorn L, Nowak N, Marshall JR, Gao AC, Ip C (2005). Microarray data mining for potential selenium targets in chemoprevention of prostate cancer. Cancer Genomics Proteomics.

[B44] Moak MA, Christensen MJ (2001). Promotion of lipid oxidation by selenate and selenite and indicators of lipid peroxidation in the rat. Biol Trace Elem Res.

[B45] Christensen MJ, Nartey ET, Hada AL, Legg RL, Barzee BR (2007). High selenium reduces NF-kappaB-regulated gene expression in uninduced human prostate cancer cells. Nutr Cancer.

[B46] Weber KS, Setchell KDR, Lephart ED (2001). Maternal and perinatal brain aromatase: effects of dietary soy phytoestrogens. Brain Res.

[B47] Wycherly BJ, Moak MA, Christensen MJ (2004). High dietary intake of sodium selenite induces oxidative DNA damage in rat liver. Nutr Cancer.

[B48] Cho SD, Jiang C, Malewicz B, Dong Y, Young CYF, Kang K-S, Lee Y-S, Ip C, Lu J (2004). Methyl selenium metabolites decrease prostate-specific antigen expression by inducing protein degradation and suppressing androgen-stimulated transcription. Mol Cancer Ther.

[B49] Dong Y, Lee SO, Zhang H, Marshall J, Gao AC, Ip C (2004). Prostate specific antigen expression is down-regulated by selenium through disruption of androgen receptor signaling. Cancer Res.

[B50] Dong Y, Zhang H, Gao AC, Marshall JR, Ip C (2005). Androgen receptor signaling intensity is a key factor in determining the sensitivity of prostate cancer cells to selenium inhibition of growth and cancer-specific biomarkers. Molecular cancer therapeutics.

[B51] Zhao H, Whitfield ML, Xu T, Botstein D, Brooks JD (2004). Diverse effects of methylseleninic acid on the transcriptional program of human prostate cancer cells. Molecular biology of the cell.

[B52] Chun JY, Nadiminty N, Lee SO, Onate SA, Lou W, Gao AC (2006). Mechanisms of selenium down-regulation of androgen receptor signaling in prostate cancer. Mol Cancer Ther.

[B53] Lee SO, Yeon Chun J, Nadiminty N, Trump DL, Ip C, Dong Y, Gao AC (2006). Monomethylated selenium inhibits growth of LNCaP human prostate cancer xenograft accompanied by a decrease in the expression of androgen receptor and prostate-specific antigen (PSA). Prostate.

[B54] Bektic J, Berger AP, Pfeil K, Dobler G, Bartsch G, Klocker H (2004). Androgen receptor regulation by physiological concentrations of the isoflavonoid genistein in androgen-dependent LNCaP cells is mediated by estrogen receptor beta. Eur Urol.

[B55] Fritz WA, Wang J, Eltoum IE, Lamartiniere CA (2002). Dietary genistein down-regulates androgen and estrogen receptor expression in the rat prostate. Mol Cell Endocrinol.

[B56] Lamartiniere CA, Cotroneo MS, Fritz WA, Wang J, Mentor-Marcel R, Elgavish A (2002). Genistein chemoprevention: timing and mechanisms of action in murine mammary and prostate. J Nutr.

[B57] Lund TD, Munson DJ, Adlercreutz H, Handa RJ, Lephart ED (2004). Androgen receptor expression in the rat prostate is down-regulated by dietary phytoestrogens. Reprod Biol Endocrinol.

[B58] Bennink MR, Om AS, Miyagi Y (1999). Inhibition of colon cancer (CC) by soy flour but not by genistin or a mixture of isoflavones. FASEB J.

[B59] Rao CV, Wang CX, Simi B (1997). Enhancement of experimental colon cancer by genistein. Cancer Res.

[B60] Yellayi S, Zakroczymski MA, Selvaraj V, Valli VE, Ghanta V, Helferich WG, Cooke PS (2003). The phytoestrogen genistein suppresses cell-mediated immunity in mice. J Endocrinol.

[B61] Prins GS (1989). Differential regulation of androgen receptors in the separate rat prostate lobes: androgen independent expression in the lateral lobe. J Steroid Biochem.

[B62] El-Bayoumy K, Richie JP, Boyiri T, Komninou D, Prokopczyk B, Trushin N, Kleinman W, Cox J, Pittman B, Colosimo S (2002). Influence of selenium-enriched yeast supplementation on biomarkers of oxidative damage and hormone status in healthy adult males: a clinical pilot study. Cancer Epidemiol Biomarkers Prev.

[B63] Crameri A, Biondi E, Kuehnle K, Lutjohann D, Thelen KM, Perga S, Dotti CG, Nitsch RM, Ledesma MD, Mohajeri MH (2006). The role of seladin-1/DHCR24 in cholesterol biosynthesis, APP processing and Abeta generation in vivo. EMBO J.

[B64] Di Stasi D, Vallacchi V, Campi V, Ranzani T, Daniotti M, Chiodini E, Fiorentini S, Greeve I, Prinetti A, Rivoltini L, Pierotti MA, Rodolfo M (2005). DHCR24 gene expression is upregulated in melanoma metastases and associated to resistance to oxidative stress-induced apoptosis. Int J Cancer.

[B65] Lu X, Kambe F, Cao X, Yoshida T, Ohmori S, Murakami K, Kaji T, Ishii T, Zadworny D, Seo H (2006). DHCR24-knockout embryonic fibroblasts are susceptible to serum withdrawal-induced apoptosis because of dysfunction of caveolae and insulin-Akt-Bad signaling. Endocrinology.

[B66] Wu C, Miloslavskaya I, Demontis S, Maestro R, Galaktionov K (2004). Regulation of cellular response to oncogenic and oxidative stress by Seladin-1. Nature.

[B67] Lai L, Tan TMC (2002). Role of glutathione in the multidrug resistance protein 4 (MRP4/ABCC4)-mediated efflux of cAMP and resistance to purine analogues. Biochem J.

[B68] Kim SO, Xu Y, Katz S, Pelech S (2000). Cyclic GMP-dependent and -independent regulation of MAP kinases by sodium nitroprusside in isolated cardiomyocytes. Biochim Biophys Acta.

[B69] Gao S, Liu G-Z, Wang Z (2004). Modulation of androgen receptor-dependent transcription by resveratrol and genistein in prostate cancer cells. Prostate.

[B70] Suzuki K, Koike H, Matsui H, Ono Y, Hasumi M, Nakazato H, Okugi H, Sekine Y, Oki K, Ito K, Yamamoto T, Fukabori Y, Kurokawa K, Yamanaka H (2002). Genistein, a soy isoflavone, induces glutathione peroxidase in the human prostate cancer cell lines LNCaP and PC-3. Int J Cancer.

[B71] Qiao S, Tuohimaa P (2004). The role of long-chain fatty-acid-CoA ligase 3 in vitamin D3 and androgen control of prostate cancer LNCaP cell growth. Biochem Biophys Res Commun.

[B72] Waku K (1992). Origins and fates of fatty acyl-CoA esters. Biochim Biophys Acta.

[B73] Papari-Zareei M, Brandmaier A, Auchus RJ (2006). Arginine 276 controls the directional preference of AKR1C9 (rat liver 3alpha-hydroxysteroid dehydrogenase) in human embryonic kidney 293 cells. Endocrinology.

[B74] Hou YT, Lin HK, Penning TM (1998). Dexamethasone regulation of the rat 3alpha-hydroxysteroid/dihydrodiol dehydrogenase gene. Mol Pharmacol.

